# Reduced Intracranial Volume in Fabry Disease: Evidence of Abnormal Neurodevelopment?

**DOI:** 10.3389/fneur.2018.00672

**Published:** 2018-08-17

**Authors:** Giuseppe Pontillo, Sirio Cocozza, Arturo Brunetti, Vincenzo Brescia Morra, Eleonora Riccio, Camilla Russo, Francesco Saccà, Enrico Tedeschi, Antonio Pisani, Mario Quarantelli

**Affiliations:** ^1^Department of Advanced Biomedical Sciences, University “Federico II”, Naples, Italy; ^2^Department of Neurosciences and Reproductive and Odontostomatological Sciences, University “Federico II”, Naples, Italy; ^3^Nephrology Unit, Department of Public Health, University “Federico II”, Naples, Italy; ^4^Institute of Biostructure and Bioimaging, National Research Council, Naples, Italy

**Keywords:** Fabry disease, neurodevelopmental disorders, magnetic resonance imaging, brain, atrophy

## Abstract

**Introduction:** Lysosomal storage disorders (LSD) are often characterized by abnormal brain development, reflected by a reduction of intracranial volume (ICV). The aim of our study was to perform a volumetric analysis of intracranial tissues in Fabry Disease (FD), investigating possible reductions of ICV as a potential expression of abnormal brain development in this condition.

**Materials and Methods:** Forty-two FD patients (15 males, mean age 43.3 ± 13.0 years) were enrolled along with 38 healthy controls (HC) of comparable age and sex. Volumetric MRI data were segmented using SPM12 to obtain intracranial tissue volumes, from which ICV values were derived.

**Results:** Mean ICV of FD patients was 8.1% smaller compared to the control group (*p* < 5·10^−5^). Unlike what typically happens in neurodegenerative disorders, no significant differences emerged when comparing between the two groups the fractional volumes of gray matter, white matter and CSF (i.e., normalized by ICV), consistent with a harmonious volumetric reduction of intracranial structures.

**Discussion:** The present results suggest that in FD patients an abnormality of brain development is present, expanding the current knowledge about central nervous system involvement in FD, further emphasizing the importance of an early diagnosis.

## Introduction

Fabry disease (FD) is a rare, X-linked disorder characterized by a progressive accumulation of globotriaosylceramide (Gb3) and related glycosphingolipids in different cells (1, 2). In contrast to many other lysosomal storage disorders (LSD) (3, 4), FD is regarded as a clinical adult-onset condition, with most patients remaining asymptomatic in the first years of life and then manifesting the characteristic multisystemic signs of the disease (2).

The use of advanced MRI techniques has recently suggested that different mechanisms of brain damage could be present in FD, with the vision of this disorder slowly changing from a purely cerebrovascular disease to a condition characterized by a more global and multifaceted cerebral involvement (5–9).

Of note, the physiopathology of CNS alterations in FD has not yet been completely understood. Along with vascular and neurodegenerative mechanisms, both due to glycosphingolipid accumulation, the possibility that in FD, similarly to other LSD, an abnormal development of CNS could occur has never been fully investigated.

Indeed, although several studies were conducted to investigate possible changes in relative brain tissue (especially gray matter –GM–) volumes in FD (7, 8, 10), to the best of our knowledge, no study has focused on the evaluation of total intracranial volume (ICV). This latter measure represents a proxy for the maximal brain growth obtained during development and maturation (11, 12), whose changes may represent a possible indicator of neurodevelopmental anomaly.

With this knowledge, aim of our study was to perform a volumetric analysis of intracranial tissues in FD, investigating possible differences in terms of ICV between FD patients and a group of healthy controls (HC) as a possible expression of abnormal brain development in this condition.

## Materials and methods

### Participants

Brain MRI studies of 42 patients with genetically proven FD, no history of any cerebrovascular events and classical phenotype of the disease were analyzed, along with those from 38 HC of comparable age and sex. For both groups exclusion criteria included a history of neurologic or major psychiatric disorders and systemic diseases that may affect the CNS (e.g., uncontrolled endocrine disorder), as well as the presence of any significant cerebral focal lesion on brain MRI.

Demographic and clinical information of all the subjects included in the analysis are listed in Table 1, while genetic findings are reported in Table s1 of the Supplementary Materials.

**Table 1:** Participants' demographic and clinical variables.

	**HC**	**FD**
Age (mean ±*SD*)	43.2 ± 14.5	43.3 ± 13.0
Sex (F–M)	23–15	27–15
ERT	n/a	31/42
ERT duration (mean ±*SD*)	n/a	51.8 ± 53.4
Hypertension	n/a	11/42
Arrhythmia	n/a	2/42
Left ventricular hypertrophy	n/a	31/42
Renal failure	n/a	14/42
Proteinuria	n/a	22/42
Neuropathy	n/a	30/42
Acroparesthesia	n/a	29/42
Gastrointestinal involvement	n/a	12/42
Cornea verticillata	n/a	33/42
Angiocheratoma	n/a	36/42

*ERT, enzyme replacement therapy; FD, Fabry disease; HC, healthy control; n/a, not applicable. Ages are expressed in years, while ERT duration is expressed in months. Renal failure considered present when the estimated glomerular filtration rate of the patient was <90 mL/min, while proteinuria was considered present when the patient scored a value >150 mg/24 h*.

### Standard protocol approvals, registrations, and patient consents

All participants gave informed written consent for use of their images for research purposes. The study was approved by the local institutional review board “Carlo Romano,” in accordance to the Declaration of Helsinki.

### MRI data acquisition

All MRI exams have been carried out on the same 3 Tesla MR scanner (Trio, Siemens Medical Systems, Erlangen, Germany). The sequences used for this study included a FLAIR sequence for assessment of White Matter Hyperintensities (WMH) and an isotropic T1w acquisition for the volumetric analysis, acquired during the same scan session. Details of the acquired sequences are reported in the Supplementary Materials.

### MRI data analysis

Brain MR scans were visually assessed for the presence of WMH by two neuroradiologists in consensus, blind to diagnosis. The load of WMH was rated in all subjects, by grading it separately in periventricular and deep hemispheric white matter (WM), with a score ranging from 0 to 3 for each location. The overall score (hereinafter Fazekas' score) was then calculated as the sum of the two values, as proposed by Fazekas et al. within a recent multicenter MR study on FD (13).

Analysis of structural T1-weighted MRI data was conducted using the Statistical Parametric Mapping (SPM12) software package (http://www.fil.ion.ucl.ac.uk/spm).

For ICV and global GM, WM, and cerebrospinal fluid (CSF) volume measures, structural data were processed using the unified segmentation tool (14); and ICV was then computed with the “tissue volumes” utility, by adding up the segmented GM, WM and CSF volumes (15).

In addition, to investigate possible changes of intracranial tissue volumes independent from ICV, fractional volumes of brain tissues were calculated as their ratio to ICV, thus obtaining fractional GM (fGM), fractional WM (fWM), and fractional CSF (fCSF) (16, 17).

Finally, to investigate possible regional GM differences between the two groups, a voxel based morphometry (VBM) analysis was carried out. With this aim, the Diffeomorphic Anatomical Registration Through Exponentiated Lie Algebra (DARTEL) algorithm (18) was employed to create a study-specific template for spatial normalization of the segmented images. The resulting flow fields created by the DARTEL procedure were then used to generate spatially normalized, Jacobian scaled, resliced (2.0-mm isotropic voxels) and smoothed (8-mm FWHM isotropic Gaussian kernel) GM maps in the standard MNI space (19).

### Statistical analysis

Differences between FD and HC groups in terms of age and sex were probed by Student *t*-test and by Chi-squared test, respectively, while differences in terms of Fazekas' score were assessed using a Mann-Whitney test. A general linear model (GLM), including age and sex as confounding covariates, was employed to probe possible differences in terms of ICV and fractional intracranial tissue volumes. Finally, correlations of volumes with Fazekas' score and clinical variables, including cardiac and renal functions, as well as ERT duration, were assessed by partial correlation analysis, correcting for age and sex. These analyses were carried out using Statistical Package for Social Science (SPSS) package (SPSS Inc., version 17.0, Chicago, IL), with a two-tailed significance level of *p* < 0.05, Bonferroni-corrected.

For the VBM analysis, differences between the two groups and correlations with the Fazekas' score and clinical variables were also assessed by means of the GLM in SPM12, including in the model age and sex, as well as ICV, to assess local differences independent from head size. In comparing the two groups, both contrasts (HC > FD and HC < FD) were probed, and differences were considered significant for *p* < 0.05, corrected for the family-wise error at cluster level.

## Results

The FD and HC groups were not significantly different for age (43.3 ± 13.0 for FD vs. 43.2 ± 14.5 for HC; *p* = 0.96), and sex (M–F = 27–15 vs. 23–15 for FD and HC, respectively; *p* = 0.73).

Among the FD patients, 20 out of 42 (47.6%) showed absence of WMH in any regions, 17 patients had a Fazekas' score of 1, 4 patients (9.5%) a score between 2 and 3, and only one patient (2.4%) a high WMH load, scoring a total Fazekas' score of 6. On the other hand, 65.8% of controls (25 out of 38 subjects) showed absence of significant WMH in any region (Fazekas' score = 0), 12 (31.6%) had a Fazekas' score of 1 and only one HC (3.1%) had a Fazekas' score of 2 (*p* = 0.07 at Mann-Whitney test).

**Figure 1:**
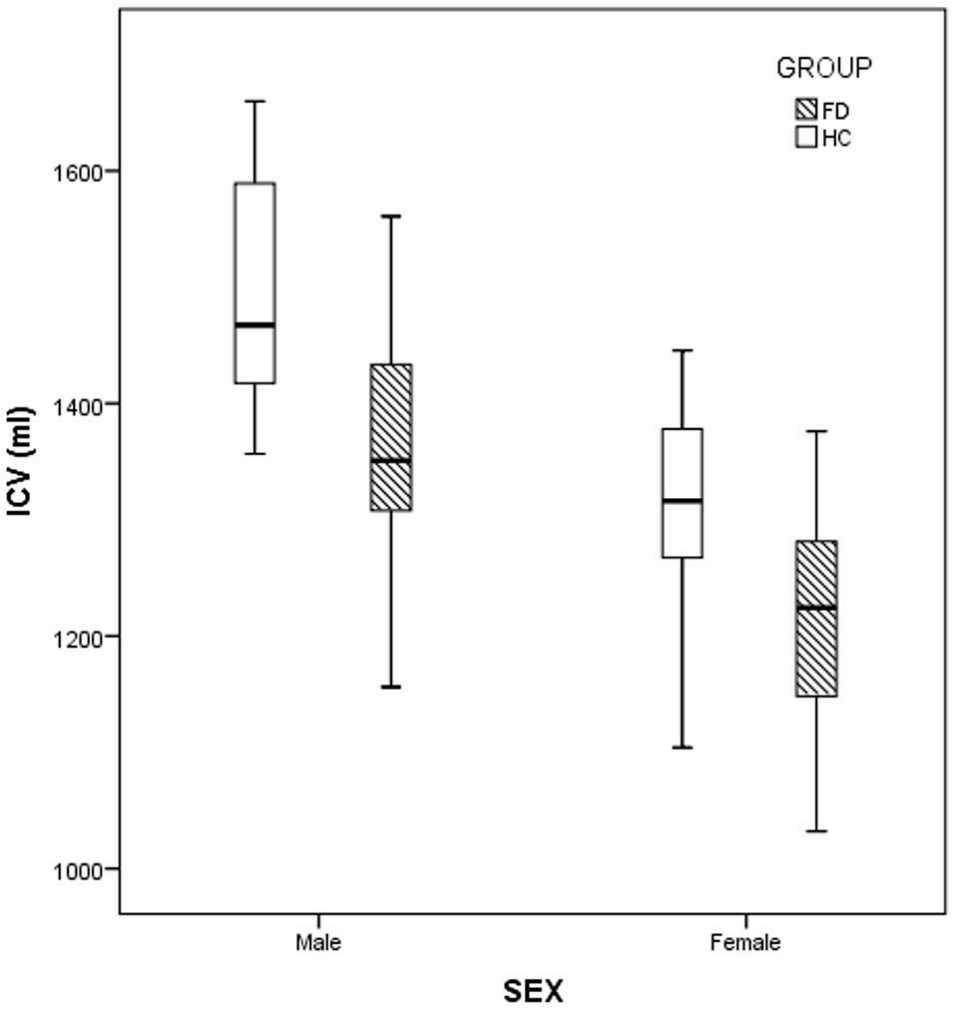
Box and whiskers plot showing the distribution of intracranial volumes (ICV) for the Fabry disease (FD) and healthy controls (HC) groups. ICV values are expressed in ml.

When evaluating possible differences in terms of ICV, FD patients showed significantly smaller volumes compared to HC, with a mean ICV that was 8.1% lower compared to the control group (1267.8 ± 121.5 ml vs. 1379.8 ± 137.2 ml in FD and HC, respectively; *p* < 5·10^−5^; 95% confidence interval of the difference = −149.1/−61.4 ml) (Figure 1).

**Figure 2:**
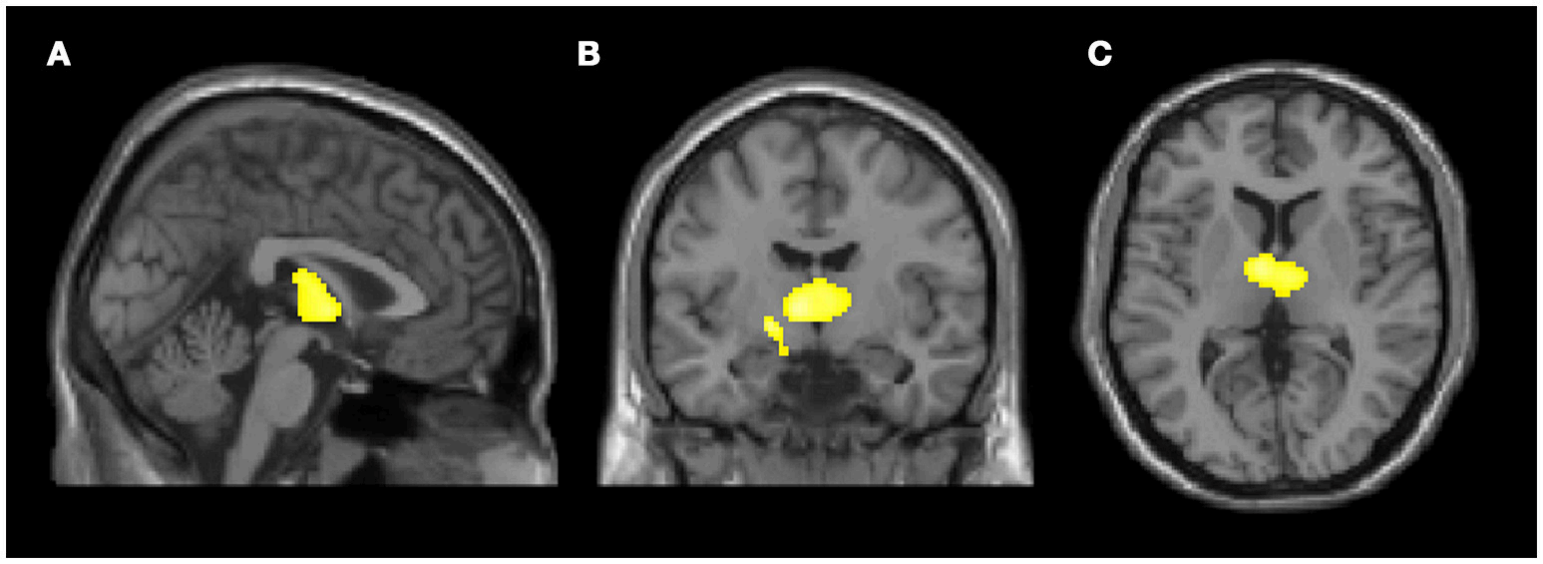
Clusters of gray matter loss in Fabry disease (FD) patients compared to healthy controls (HC). Results are displayed for *p* < 0.05 FWE-corrected at cluster level, and superimposed for anatomical reference to multi-planar reconstruction of a single subject T1-weighted volume in the standard Montreal Neurological Institute space **(A)** sagittal, **(B)** coronal, **(C)** axial. No region of increased gray matter volume was present in FD patients compared to HC.

No significant differences emerged between the two groups when comparing the fGM (50.5 ± 3.6% vs. 50.1 ± 3.9% in FD and HC, respectively; *p* = 0.41; 95% confidence interval of the difference = −0.6%/1.4%), the fWM (30.6 ± 2.0% vs. 31.4 ± 2.0% in FD and HC, respectively; *p* = 0.10; 95% confidence interval of the difference = −1.6%/0.1%) and the fCSF (18.8 ± 4.4% vs. 18.5 ± 4.0% in FD and HC, respectively; *p* = 0.86; 95% confidence interval of the difference = −1.0%/1.7%). The VBM analysis revealed only a cluster of reduced GM density in FD patients compared to HC at the level of the thalami, bilaterally, extending toward the left hippocampus (*p* = 0.001) (Figure 2; Table s2). No significant clusters of increased GM density were found in FD patients compared to HC.

Finally, when testing the relationship with WMH load and clinical variables, no significant correlation emerged for ICV, fractional intracranial tissue volumes or regional GM density.

## Discussion

We investigated volumetric alterations of intracranial structures in FD patients, demonstrating a smaller ICV in these subjects compared with HC, along with a preservation of fractional intracranial tissue volumes. These results, taken together, demonstrate a harmonious reduction of all intracranial tissue volumes, supporting the hypothesis of a possible abnormality of neural development in these patients.

ICV is defined as the sum of GM, WM, and CSF, and it is a representation of the maximal brain growth obtained during development and maturation (11, 12). Indeed, the peak of the ICV is reached early in life, such that, by the age of 6 years, total brain volume is 90% of adult volume and reaches full adult volume during adolescence (20, 21). Once ICV peaks and the skull sutures are completely fused, there is no further change in this measure, regardless of changes that may occur in brain tissues (22), which when occurring must be compensated by changes in the CSF compartment volume.

Most sphingolipidoses, and LSD in general, are characterized by the relatively prominent involvement of CNS, ranging from early developmental delay and microcephaly to more subtle neurodevelopmental deficits (23–26).

Interestingly, some studies demonstrated a smaller ICV as an evidence of neurodevelopmental defect not only in early-onset neurodevelopmental disorders but also in other genetic conditions mainly characterized by an adult-onset neurodegeneration, such as Wolfram Syndrome or even prodromal Huntington's Disease (27, 28). These findings suggest that in these diseases subtle neurodevelopmental phenomena may occur already in the preclinical phases (27, 28).

In our study, we found significantly smaller intracranial volumes in FD patients, a finding that could shed further light on the mechanisms of cerebral involvement in FD, suggesting for the first time that in these patients a neurodevelopmental abnormality may be also present. Indeed, since ICV, once determined by maximal brain growth in childhood, does not change over time, a smaller volume could reflect an incomplete growth of the brain parenchyma, as expression of a global abnormality in the process of brain development (27).

A possible explanation to this finding could be traced to the pathogenic mechanisms typical of FD. Indeed, it may be hypothesized that α-galactosidase A could play a role in the normal development of the CNS and, similarly to what happens in many other LSD (23, 24, 26), its deficiency could lead to detectable, although mild, neurodevelopmental abnormalities. This speculation, coupled to other evidences demonstrating the possibility of an early detection of clinical and subclinical manifestations in pediatric FD patients (29, 30), could further indicate that FD, once considered as an adult-onset condition, is indeed a more complex phenomenon that encompass all ages, also regarding CNS involvement. The present findings thus further stress the importance of a timely diagnosis, or even of newborn screening, and the possible advantages of an early initiation of the enzyme-replacement therapy (ERT) (31). With respect to this speculation, it should also be noted that the absence of a significant correlation with the ERT duration is in line with our hypothesis. Indeed, all FD patients included in the study started the ERT as adults, where this possible neurodevelopmental abnormality could have already developed. To test this hypothesis, however, longitudinal studies including pediatric FD patients should be performed, to further elucidate any possible relationship between ERT and ICV variations.

Along with the evaluation of ICV, we have investigated if different intracranial tissues were affected to a different extent in FD, showing no difference between FD and HC in terms of fractional intracranial tissue volumes. This finding, coupled to the abovementioned ICV reduction in these patients, indicates the presence of a harmonious volumetric reduction of all intracranial structures in FD patients, rather than a pure neurodegenerative phenomenon. Indeed, neurodegeneration usually affects GM or the entire brain parenchyma volume, determining a reduction of the corresponding fractional volumes, typically with a compensatory increase in CSF volume to maintain total ICV unchanged.

Interestingly, in the *post-hoc* VBM analysis we found two clusters of reduced GM density involving both thalami with extension to the left hippocampus, in absence of significant correlation between regional GM volume and WMH load. This finding is apparently in conflict with previous volumetric studies, which failed to prove significant regional alterations in GM volumes of FD patients (8, 9). A possible explanation to this discrepancy may lay in the segmentation method used in the present work, coupled to the larger sample examined in this work, which provides a slightly greater statistical power, that allowed to detect these subtle differences. Indeed, our analysis was conducted with a different software package (i.e., SPM12) compared to the one used in previous works (e.g., SPM8), which has proved to be one of the most accurate method for intracranial volumes assessment (15, 32). Supporting this speculation, an additional analysis performed on the same subjects with SPM8 led to no significant differences at the VBM analysis (data not shown).

Indeed, our VBM findings are also partially in line with previous studies that demonstrated hippocampal atrophy in FD patients as a possible surrogate consequence of a primitive neuronal involvement, independent from brain vasculopathy (7, 10). Moreover, similar findings were also reported in other sphingolipidoses such as Niemann-Pick disease, indicating a preferential volume reduction of subcortical gray matter structures in these conditions (33, 34). Slightly more prominent thalamic and hippocampal atrophy, in the context of a generalized harmonic reduction of the volumes of all intracranial compartments, could be the expression of an additional abnormal development of subcortical GM or, more probably, of a specific vulnerability to neurodegeneration due to selective neuronal Gb3 accumulation in these structures, which has been reported in anatomopathological studies (7, 35–37). However, longitudinal and neuropsychological studies are needed to further elucidate the physiopathological mechanisms and the clinical meaning of this finding.

It is known that in this condition height is reported to be below the US 50th percentile in young male patients, while no significant difference is described for female patients (29). This evidence could lead to the interpretation of our finding as an expression of a generalized harmonious growth defect, rather than a selective neurodevelopmental abnormality. To test this hypothesis however, we retrospectively retrieved anthropometric measures for the FD group from clinical records, which were not significantly different from the reported values of the adult population of the same geographical area for both males [median height: 170 cm (range: 158–188 cm) vs. 174 cm in the healthy population; *p* = 0.18; 95% confidence interval of the difference = −6.72/1.38] and females [median height: 160 cm (range: 145–175 cm); vs. 161 cm in the healthy population, *p* = 0.81; 95% confidence interval of the difference = −2.92/2.30] (38). Furthermore, an ancillary partial correlation analysis (corrected for age and sex) proved no significant correlations between height and ICV values (*r* = 0.225, *p* = 0.169; 95% confidence interval for *r* = −0.089/0.498) in our patients. Although these observations further support our speculation that a reduction of ICV values could be the expression of a neurodevelopmental abnormality, we could not fully test this hypothesis as anthropometric measures were not available in the HC group, so that further studies are warranted to confirm it.

In conclusion, we demonstrated that FD patients show a highly significant reduction of the ICV compared to HC, while fractional volumes of intracranial tissues are preserved in this condition. Our results further expand the current knowledge about CNS involvement in FD, suggesting that in these patients an abnormality of brain development could be present, thus emphasizing the importance of an early diagnosis of FD, and possibly of an early ERT initiation.

Further studies are needed to understand if this feature of the disease is part of the spectrum of more generalized growth defect encountered in this pathology, or it represents an independent phenomenon.

## Author contributions

GP and SC: conception, organization, and execution of the research, writing of the manuscript; AB: organization and execution of the research, writing, and review of the manuscript; VBM: organization and execution of the research, writing of the manuscript; ER: conception and execution of the research, review of the manuscript; CR: conception of the research, writing, and review of the manuscript; FS and ET: organization and execution of the research, review of the manuscript; AP: writing, review, and critique of the manuscript; MQ: design, execution, review, and critique of the statistical analysis, review, and critique of the manuscript.

### Conflict of interest statement

SC received fees for speaking from Genzyme. AB received travel grants for participation in scientific meetings on contrast media by Bracco and user meetings by General Electric. VBM reports personal fees from Novartis, Biogen, Genzyme, Teva, Almirall, Bayer, and Merck. ER received travel grant and honoraria for serving as consultant to Genzyme and Shire. ET received fees for speaking from Scientific Press, Ars Educandi, and Shire Italy. AP received travel grant and honoraria for serving as consultant to Genzyme, Shire and Amicus. The remaining authors declare that the research was conducted in the absence of any commercial or financial relationships that could be construed as a potential conflict of interest.

## Abbreviations

LSD: Lysosomal Storage Disorder
ICV: Intracranial Volume
FD: Fabry Disease
HC: Healthy Control
WMH: White Matter Hyperintensities
fGM: fractional Gray Matter
fWM: fractional White Matter
fCSF: fractional Cerebro-Spinal Fluid
ERT: Enzyme-Replacement Therapy.
