# Supplementary material for: Reduced Intracranial Volume in Fabry Disease: Evidence of Abnormal Neurodevelopment?
Source: Front Neurol. 2018 Aug 17;9:672. doi: 10.3389/fneur.2018.00672 (PMC6107697; doi:10.3389/fneur.2018.00672)
Supplement: Supplementary file 1 [file Data_Sheet_1.doc]

**REDUCED INTRACRANIAL VOLUME IN FABRY DISEASE: EVIDENCE OF ABNORMAL NEURODEVELOPMENT?**

*Giuseppe Pontillo**, MD ^1#^, Sirio Cocozza**, MD ^1#*^, Arturo Brunetti, MD ^1^, Vincenzo Brescia Morra, MD ^2^, Eleonora Riccio, MD, PhD ^3^, Camilla Russo, MD ^1^, Francesco Saccà,* *MD ^2^, Enrico Tedeschi, MD ^1^**, Antonio Pisani,* *MD, PhD ^3^, Mario Quarantelli, MD ^4^, on behalf of the AFFINITY study group*

^1^ Department of Advanced Biomedical Sciences, University “Federico II”, Naples, Italy

^2^ Department of Neurosciences and Reproductive and Odontostomatological Sciences,

University “Federico II”, Naples, Italy

^3^ Department of Public Health, Nephrology Unit, University “Federico II”, Naples, Italy

^4^ Institute of Biostructure and Bioimaging, National Research Council, Naples, Italy

*^#^* These authors equally contributed to this work

**MRI data acquisition**

All MRI exams have been carried out on the same 3 Tesla MR scanner (Trio, Siemens Medical Systems, Erlangen, Germany). Brain scans included a three-dimensional Fluid-Attenuated Inversion Recovery (FLAIR) sequences, acquired with the following parameters: TR = 6000 ms; TE = 396 ms; TI = 2200 ms; Flip Angle = 120°; voxel size = 1x1x1 mm^3^; number of slices = 160; sagittal orientation, and a structural T1w volumes acquired using a three-dimensional magnetization-prepared rapid gradient-echo sequence (MPRAGE; axial planes; TR=1900 ms; TE=3.4 ms; TI=900 ms; Flip Angle=9°; voxel size=1x1x1 mm^3^; number of slices=160).

**Table s1**

|  | **Sex** | **Age** | **Mutation** | **Residual Enzyme Activity** |
| --- | --- | --- | --- | --- |
| *Patient #1* | M | 55 | A.288D | 0.3 |
| *Patient #2* | M | 43 | G740A | 0.2 |
| *Patient #3* | M | 38 | G740A | 0.2 |
| *Patient #4* | F | 33 | c.1133C>T | 2.5 |
| *Patient #5* | F | 68 | c.67T>G | 3.4 |
| *Patient #6* | M | 49 | p.R356W | 1.8 |
| *Patient #7* | M | 34 | c.67T>G | 0.2 |
| *Patient #8* | M | 50 | c.1066C>T | 0 |
| *Patient #9* | F | 56 | p.R356W | 3.7 |
| *Patient #10* | F | 36 | p.R356W | 3.5 |
| *Patient #11* | F | 30 | c.901C>G | 3.9 |
| *Patient #12* | F | 25 | A.288D | 6.4 |
| *Patient #13* | M | 52 | p.Trip162X | 3.1 |
| *Patient #14* | F | 26 | c.1066C>T | 4.3 |
| *Patient #15* | M | 56 | c.1066C>T | 2.7 |
| *Patient #16* | M | 54 | c.1066C>T | 0 |
| *Patient #17* | M | 47 | c.1066C>T | 2.3 |
| *Patient #18* | F | 51 | c.1021dupG | 1.9 |
| *Patient #19* | F | 45 | c.1021dupG | 2.3 |
| *Patient #20* | F | 28 | c.1021dupG | 4.4 |
| *Patient #21* | M | 43 | c.1133C>T | 0 |
| *Patient #22* | F | 59 | c.1066C>T | 2.1 |
| *Patient #23* | F | 46 | p.R356W | 4.7 |
| *Patient #24* | F | 20 | IVS4+5G>T | 4.7 |
| *Patient #25* | F | 49 | IVS4+5G>T | 2.7 |
| *Patient #26* | M | 45 | IVS4+5G>T | 0.6 |
| *Patient #27* | F | 35 | c.680G>C | 4.1 |
| *Patient #28* | F | 32 | c.680G>C | 2.9 |
| *Patient #29* | F | 62 | c.680G>C | 2.8 |
| *Patient #30* | F | 29 | c.901C>G | 0.2 |
| *Patient #31* | F | 57 | c.901C>G | 3.0 |
| *Patient #32* | F | 33 | c.424T>C | 6.3 |
| *Patient #33* | F | 48 | c.1066C>T | 3.7 |
| *Patient #34* | F | 65 | G740A | 2.5 |
| *Patient #35* | M | 35 | c.352C>T | 3.4 |
| *Patient #36* | F | 38 | c.508G>A | 4.8 |
| *Patient #37* | F | 19 | c.901C>G | 3.0 |
| *Patient #38* | M | 46 | c.901C>G | 0.8 |
| *Patient #39* | F | 30 | c.901C>G | 1.5 |
| *Patient #40* | F | 34 | c.901C>G | 1.5 |
| *Patient #41* | F | 73 | c.1066C>T | 2.3 |
| *Patient #42* | M | 44 | c.1066C>T | 0 |

**Table s1:** List of mutations and residual enzyme activity for all FD patents included in the study.

Table s2

| **Cluster**  **Volume (ml)** | **T** |  | **^p^_FWE-corr_** | **X** | **Y** | **Z** |  |  |
| --- | --- | --- | --- | --- | --- | --- | --- | --- |
| 7.8 | 5.01 |  | 0.029 | -14 | -2 | -8 |  | Left Pallidum |
|  | 4.38 |  | 0.181 | -6 | -6 | 2 |  | Left Thalamus |
|  | 4.03 |  | 0.483 | 2 | -14 | 14 |  | Right Thalamus |

**Table s2: Results of the VBM analysis**

Cluster of reduced GM volume in FD patients compared to HC.

Results are reported for p<0.05, FWE-corrected at cluster level, along with the corresponding maximum T values. For the three local maxima, p-values corrected at voxel level are also reported (FWE-corrected). No significant differences emerged when testing the FD > HC contrast.

Coordinates refers to mm from the anterior commissure in MNI space, with anatomical labeling according to (1).

**References**

1. Tzourio-Mazoyer N, Landeau B, Papathanassiou D, et al. Automated anatomical labeling of activations in SPM using a macroscopic anatomical parcellation of the MNI MRI single-subject brain. NeuroImage. 2002 Jan;15(1):273-289.
